# Association between living arrangement and quality of life for older adults in the community*

**DOI:** 10.1590/1518-8345.4051.3401

**Published:** 2021-01-08

**Authors:** Alisson Fernandes Bolina, Mayssa da Conceição Araújo, Vanderlei José Haas, Darlene Mara dos Santos Tavares

**Affiliations:** 1 Universidade de Brasília, Departamento de Enfermagem, Brasília, DF, Brazil.; 2 Universidade Federal do Triângulo Mineiro, Uberaba, MG, Brazil.

**Keywords:** Family Characteristics, Aged, Quality of Life, Family, Aging, Geriatric Nursing, Composição Familiar, Idoso, Qualidade de Vida, Família, Envelhecimento, Enfermagem Geriátrica, Composición Familiar, Anciano, Calidad de Vida, Familia, Envejecimiento, Enfermería Geriátrica

## Abstract

**Objective:**

to compare the sociodemographic and economic characteristics of the older adults in the community according to the living arrangement and to verify the association between the type of living arrangement and the quality of life scores.

**Method:**

a cross-sectional epidemiological study conducted with 796 older adults in the community. To assess quality of life (dependent variable), network and social support (adjustment variable), validated and applied chi-square tests, descriptive statistical analysis, multiple comparison analysis (ANOVA) and multiple linear regression model (p<0.05) were used.

**Results:**

the older adults who lived only with their spouses had better quality of life scores in all domains and facets, except in the death and dying domain, which did not show any significant difference. The lowest scores for quality of life were identified in the groups with the presence of children and, exceptionally, in the domain of social relationships and, in the facets death and dying and intimacy, those who lived alone had worse assessments. In the adjusted model, there was an association between the type of living arrangement and the different domains and facets of quality of life.

**Conclusion:**

living arrangement was associated with quality of life scores for older adults in the community, even after adjusting for the gender, age, number of morbidities, and social support variables.

## Abstract (pt)

**Objetivo:**

comparar as características sociodemográficas e econômicas de idosos da comunidade segundo o arranjo domiciliar e verificar associação entre o tipo de arranjo domiciliar e os escores de qualidade de vida.

**Método:**

estudo epidemiológico transversal, com 796 idosos da comunidade. Para avaliar qualidade de vida (variável dependente), rede e apoio social (variável de ajuste), foram utilizados instrumentos validados e aplicados teste qui-quadrado, análise estatística descritiva, análise de comparações múltiplas (ANOVA) e modelo de regressão linear múltipla (p<0,05).

**Resultados:**

os idosos que residiam somente com os cônjuges apresentaram melhores escores de qualidade de vida em todos os domínios e facetas, exceto no domínio morte e morrer, que não evidenciou diferença significativa. Os menores escores de qualidade de vida foram identificados nos grupos com a presença dos filhos, sendo que, excepcionalmente no domínio relações sociais e nas facetas morte e morrer e intimidade, aqueles que viviam sozinhos tiveram piores avaliações. No modelo ajustado, verificou-se associação entre o tipo de arranjo domiciliar e os diferentes domínios e facetas de qualidade de vida.

**Conclusão:**

o arranjo domiciliar apresentou associação com escores de qualidade de vida de idosos da comunidade, mesmo após ajuste para as variáveis sexo, idade, número de morbidades e apoio social.

## Abstract (es)

**Objetivo:**

comparar las características sociodemográficas y económicas de los adultos mayores de la comunidad según la conformación domiciliaria y verificar la asociación entre el tipo de conformación domiciliaria y los puntajes de calidad de vida.

**Método:**

estudio epidemiológico transversal con 796 ancianos de la comunidad. Para evaluar la calidad de vida (variable dependiente), la red y el apoyo social (variable de ajuste), se utilizaron instrumentos validados y aprobados, pruebas de chi-cuadrado, análisis estadístico descriptivo, análisis de comparación múltiple (ANOVA) y modelo de regresión lineal múltiple (p<0,05).

**Resultados:**

los adultos mayores que vivían solamente con sus cónyuges obtuvieron mejores puntajes de calidad de vida en todos los dominios y facetas, excepto en el dominio muerte y morir, que no mostró diferencia significativa. Los puntajes más bajos en calidad de vida se identificaron en los grupos con presencia de hijos y, de manera excepcional en el dominio de las relaciones sociales y en las facetas muerte y morir e intimidad, aquellos que vivían solos tuvieron peores evaluaciones. En el modelo ajustado, se verificó asociación entre el tipo de conformación domiciliaria y los diferentes dominios y facetas de calidad de vida.

**Conclusión:**

la conformación domiciliaria se asoció con puntajes de calidad de vida de los adultos mayores de la comunidad, incluso después del ajuste por las variables sexo, edad, número de morbilidades y apoyo social.

## Introduction 

Population aging has caused changes in the structure of families that raise reflections on the sustainability of the traditional models of family care for the older adults. Although the increase in life expectancy has increased the number of surviving generations, there is currently less likelihood of multi-generational cohabitation in the same family. In addition, the decline in fertility (reduction in the number of young people), changes in gender patterns (insertion of women in the labor market), and the weakening of the social representation of the older adults in contemporary society compromise the family’s ability to provide care for the older adults^(^ 1 ^)^.

In this scenario, new configurations of living arrangements for the older adults^(^ 2 ^)^ have been discussed, and this issue is an object for pressing concern on the world population aging agenda^(^ 1 ^)^. In this study living arrangement is understood as the composition of the individuals who reside in the same physical space (home), considering the identity of cohabitants (spouse, children, grandchildren, caregiver, and others). This is an operational definition also adopted in other research studies in Brazil^(^ 3 ^)^ and in the world^(^ 4 ^)^.

Although the majority of the older adults in the world lives with someone, it is important to mention the recent exponential increase in single-person homes^(^ 5 ^)^. Specifically in Brazil, according to data from the National Health Survey, 15.3% of the older adults lived alone in 2013^(^ 6 ^)^. Although this type of arrangement represents an achievement and a desire of the older adult to live alone, on the other hand, it can be a risk factor for social isolation, with harms to mental health and difficulties in accessing the health services^(^ 3 ^-^ 7 ^)^.

Several scholars in the field highlight the role of the family as a source of emotional, instrumental and financial support, which positively impacts on mental health and on the degree of satisfaction with life of the older adults^(^ 8 ^-^ 9 ^)^. In a study carried out in China, the older adults who lived with their families were more likely to receive financial resources and emotional support from their relatives compared to those who lived alone^(^ 10 ^)^.

On the other hand, the co-residence of the older adult with the family by itself does not guarantee support in times of need^(^ 3 ^)^, since the family members may not be prepared to deal with the specificities of aging, especially due to the difficulty in offering instrumental support in daily life^(^ 10 ^)^.

From this perspective, each type of living arrangement has potentials and weaknesses that raise the need to further investigate the association between social isolation, mental health, and the well-being of the older adult^(^ 7 ^)^. All of these factors directly affect Quality of Life (QoL)^(^ 7 ^)^, herein understood as “[...]the individual’s perception of their position in life, in the context of the culture and value system in which they live and in relation to their goals, expectations, standards and concerns”^(^ 11 ^)^.

In the gerontological literature, a number of studies have found that the older adults who live with adult children^(^ 5 ^,^ 12 ^)^, live alone^(^ 5 ^,^ 7 ^)^ or with other family members (with no presence of the spouse)^(^ 7 ^,^ 13 ^)^ have showed harms in QoL. However, these studies did not adjust for social support, which will be analyzed in this study.

Regardless of the type of living arrangement, social support promotes significant engagement and emotional support, as well as it avoids social isolation^(^ 14 ^)^, therefore, being able to play a mediating role between the living arrangement and QoL. Results of a research study conducted with Chinese older adults living alone suggest that social support can mitigate the negative effects on QoL^(^ 9 ^)^. In another study also carried out in China, urban older adults who lived alone were the most vulnerable to the worst health-related quality of life scores, and social interaction reduced this negative effect^(^ 14 ^)^. However, so far, no research study has been identified to verify the association between living arrangement and the QoL domains and facets of urban older adults in the community through the application of a specific instrument for this age group and adjustments for the potential confounding variables, such as social support.

Given the above, some questions emerge: Which type of living arrangement contributes the most to the QoL of urban older adults in the community? Does the relationship between the living arrangement and the QoL scores of the older adults remain the same after controlling for social support? It should be mentioned that the configuration of living arrangement in Brazil does not depend exclusively on the will of the older adults or of their families, but involves a variety of historical, sociocultural, political, economic and demographic aspects, which can favor or harm QoL^(^ 15 ^)^.

Therefore, it is believed that investigating the relationship between different types of contemporary living arrangements and the QoL of the older adult population in a Brazilian municipality will contribute to elucidating the potential and specific challenges of each housing context, which, in turn, need be considered by researchers, educators, health and social care professionals and, above all, by public policy-makers. As a member of the family health team, the nurse has a primary role in caring for the older adults in the community, with the housing context and the social support network aspects that should be analyzed during the nursing consultation.

This study has the following objectives: to compare the sociodemographic and economic characteristics of the community’s older adults according to living arrangement; and to verify the association between the type of living arrangement and the quality of life scores.

## Method

A quantitative approach study, of the home survey type, observational, analytical and cross-sectional, developed in the urban area of the city of Uberaba, located in the state of Minas Gerais.

Older adults (60 years old or more) living in the urban area of that municipality took part in the study. The sample was defined based on procedures for multi-stage cluster sampling. The sample size calculation considered a determination coefficient in a multiple linear regression model with five predictors, with a level of significance or type I error of and type II error of β = 0,1 , therefore resulting in an *a priori* statistical power of 90%. The values described above were inserted in the PASS (Power Analysis and Sample Size) application, version 13, obtaining *n=813*.

The inclusion criteria were the following: being 60 years old or older and living in the urban area of the municipality of Uberaba-MG. Institutionalized older adults were excluded; as well as those with communication problems (deafness not corrected by hearing aids and severe speech disorders), those who were not found after three attempts by the interviewer, and those who presented cognitive decline associated with a final score ≥ 6 points in the PFEFFER Functional Activities Questionnaire. Based on such criteria, 17 older adults were excluded: 12 for having cognitive decline and a score > 6 points in the questionnaire, and five that did not have complete data in relation to the outcome variables. The final sample of the study comprised 796 older adults.

Data collection was carried out at the older adults’ homes from May 2017 to June 2018, by means of direct interviews using the instruments described below. Initially, the older adult’s cognitive assessment was carried out through the application of the Mini Mental State Examination (MMSE), translated and validated in Brazil^(^ 16 ^)^ and, in a positive screening for cognitive decline, the PFEFFER Functional Activities Questionnaire^(^ 17 ^)^ was applied to the older adults. This scale has 11 questions with a maximum score of 33 points and allows verifying the presence and severity of cognitive decline through the assessment of functionality and the need for assistance from other people. In the present study, for situations in which the PFEFFER result was less than six points, it was defined that the interview would be carried out with the older adult and, if necessary, the information could be complemented by the informant.

To collect the explanatory and adjustment variables, a structured form with the following information was used: (1) socioeconomic: gender (male and female), age (numerical variable) and/or age group, in years old (60 ├70, 70 ├80, and 80 or more); schooling, in years of study (None, 1 ├ 4, 4 ├ 8, 8 ├ 11, and 11 and more); individual monthly income, in minimum wages (without income, < 1, 1, 1 ┤3, > 3); and (2) number of self-reported morbidities.

Another adjustment variable was network and social support, assessed by the scale originally prepared by the Medical Outcomes Study (MOS)^(^ 18 ^)^, translated and validated for Brazil^(^ 19 ^)^. The scale consists of five dimensions (material support, affective support, emotional support, information, and social interaction), assessed by means of questions in which the older adults indicate how often they consider each type of support available, in case of need: 1 (never); 2 (rarely); 3 (sometimes); 4 (almost always), and 5 (always). Based on the answers, the scale generates a score, and the higher the score, the better the social support^(^ 19 ^)^.

To assess the living arrangement (preferred predictor variable), the older adult was asked “Do you live at your home?” and offered the following answer options: alone; only with some professional caregiver; only with the spouse; with others of your generation with or without a spouse; with children with or without a spouse; with grandchildren with or without a spouse; other arrangements. Based on the answers, the study sample was re-categorized into six groups according to the type of living arrangement: they lived only with their spouse; alone; only with children; with spouse and others; with children and others; and had other types of arrangements.

QoL – response or outcome variable – was measured using two instruments validated in Brazil: the World Health Organization Quality of Life Bref (WHOQoL-Bref), composed by four domains (physical, psychological, social relations and environment)^(^ 20 ^)^, and the World Health Organization Quality of Life Old (WHOQoL-Old), consisting of six facets (functioning of the senses; autonomy; past, present and future activities; social participation; death and dying; and intimacy)^(^ 21 ^)^. It is noteworthy that the instruments are complementary for assessing QoL, as the WHOQoL-Bref measures this variable in a generic way and the WHOQoL-Old is specific for older adults. The scores were measured using syntax and vary from 0 to 100, where the highest value represents the best QoL.

The collected data were processed in spreadsheets using the Excel^®^ program, in two databases for consistency assessment. Then, they were exported to the Statistical Package for the Social Sciences (SPSS), version 12.0 in order to proceed with data analysis.

Descriptive statistical analysis was conducted by means of the distribution of absolute frequencies and percentages for the categorical variables and means and standard deviations for the numerical variables. The chi-square test was used to compare sociodemographic and economic characteristics according to the living arrangement. To meet the second objective, the analysis of multiple comparisons (ANOVA) was carried out first, using the Bonferroni adjustment criterion. The adjustment for the gender, age, number of morbidities, and social support variables was made using the multiple linear regression model. In all the analyses, the level of significance (α) was 5%, and the tests considered significant when *p* ≤ α.

The study was approved by the Ethics Committee for Research with Human Beings of the Federal University of Triângulo Mineiro, Uberaba-MG, under protocol number 2,053,520.

## Results

The final sample of the study comprised 796 older adults, divided into six groups according to the type of living arrangement: 22.2% (n =177) lived only with their spouse; 18.5% (n=147) alone; 16.8% (n=134) only with children; 20.0% (n=159) with the spouse and others; 12.8% (n=102) with children and others; and 9.7% (n=77) had other types of living arrangement.

As shown in Table 1, the highest percentage of older adults living with a spouse and others was male, unlike the other groups, where women prevailed (*p*<0,001). It was verified that the highest percentage of older adults who lived only with their spouse and with their spouse and others was in the age range of 60 ├70 years old. The other types of arrangement concentrated older adults aged 70 ├80 years old (*p*<0.001), with emphasis on those who lived alone. With regard to schooling, the highest percentage had 4 ├8 years of study in all types of living arrangement. However, higher proportions of older adults with lower levels of schooling (none and 1 ├ 4 years of study) were found in the arrangements only children/children and others/alone when compared to the others (*p*=0.001). With regard to individual monthly income, older adults prevailed who received up to one minimum wage in all types of living arrangement, followed by those with an income of 1┤3 minimum wages (*p*<0.001).

**Table 1:** Comparison of the absolute frequencies and percentages of the sociodemographic and economic variables according to the older adults' living arrangements. Uberaba, MG, Brazil, 2017-2018

Variables	Spouse only		Alone		Children only		Spouse and others		Children and others		Other types		χ2*	*p* ^†^
	N	%	N	%	N	%	N	%	n	%	N	%		
Gender														
Female	91	51.4	95	64.6	112	83.6	76	47.8	86	84.3	70	90.9	96.1	<0.001
Male	86	48.6	52	35.4	22	16.4	83	52.2	16	15.7	7	9.1		
Age group (in years old)														
60├ 70	77	43.5	39	26.5	40	29.9	83	52.2	28	27.5	24	31.2	53.5	0.001
70├ 80	76	42.9	72	49.0	55	41.0	61	38.4	39	38.2	32	41.6		
80 and over	24	13.6	36	24.5	39	29.1	15	9.4	35	34.3	21	27.3		
Schooling (in years)														
None	24	13.6	29	19.7	34	25.4	15	9.4	23	22.5	9	11.7	37.7	0.001
1├ 4	28	15.8	34	23.1	36	26.9	37	23.3	25	24.5	16	20.8		
4├ 8	77	43.5	59	40.1	40	29.9	60	37.7	40	39.2	31	40.3		
8 and over	48	27.1	25	17.0	24	17.9	47	29.6	14	13.7	21	27.3		
Individual monthly income^‡^														
No income	23	13.0	0	0	2	1.5	16	10.1	1	1.0	1	1.3	58.5	0.001
Up to 1 salary	76	42.9	74	50.3	75	56.0	79	49.7	58	56.9	41	53.2		
1 ┤3	63	35.6	69	46.9	50	37.3	51	32.1	39	38.2	31	40.3		
> 3	15	8.5	4	2.7	7	5.2	13	8.2	4	3.9	4	5.2		

* Chi-square test;

† p-value;

‡ Minimum wage in force and effect during the data collection period: 2017 (R$ 937.00) and 2018 (R$ 954.00)

When comparing the QoL scores with the types of living arrangements shown in Table 2, it appears that the older adults who lived only with their spouses, in general, had better scores for quality of life in all the domains and facets of QoL (*p*<0.05), except in the death and dying domain, which showed no significant difference between the groups. It is noteworthy that the lowest QoL scores were found in the groups of children and others or only children, being that, exceptionally in the social relationships domain and in the facets death and dying and intimacy, those who lived alone had the worst assessments.

**Table 2:** Comparison of the QoL* scores in the WHOQoL-Bref domains and WHOQoL-Old facets according to the living arrangement of the older adults. Uberaba, MG, Brazil, 2017-2018

Domains/Facets QoL*	Spouse only	Alone	Children only	Spouse and others	Children and others	Other types	F^†^	*p* ^‡^
	Mean (SD±)	Mean (SD±)	Mean (SD±)	Mean (SD±)	Mean (SD±)	Mean (SD±)		
*WHOQoL-Bref*								
Physical	68.1 (17.4)	64.6 (16.9)	63.2 (17.2)	65.6 (16.8)	61.3 (15.5)	62.9 (17.5)	2.75	0.018
Psychological	73.8 (14.1)	68.0 (15.7)	68.7 (15.8)	72.0 (13.5)	66.7 (13.5)	69.5 (13.2)	4.90	0.001
Social relationships	73.4 (14.9)	66.0 (17.2)	67.4 (16.7)	72.9 (14.6)	69.3 (13.8)	67.1 (17.1)	5.97	0.001
Environment	68.5 (13.3)	63.8 (14.0)	65.9 (13.3)	67.2 (13.0)	63.5 (12.8)	64.6 (13.9)	3.17	0.008
*WHOQoL-Old*								
Functioning of the senses	78.0 (20.8)	73.1 (22.5)	67.7 (24.0)	76.9 (22.1)	68.7 (24.1)	76.0 (21.6)	5.06	0.001
Autonomy	73.6 (13.6)	69.9 (16.5)	65.9 (17.0)	69.7 (15.6)	67.0 (15.5)	67.1 (14.0)	4.80	0.001
Past/Present/Future activities	72.9 (13.2)	66.2 (16.9)	68.6 (16.1)	70.5 (13.5)	65.9 (13.4)	67.6 (14.9)	5.07	0.001
Social participation	71.7 (15.5)	66.0 (17.0)	66.7 (18.0)	67.2 (16.5)	62.8 (15.3)	66.4 (16.0)	4.30	0.001
Death and dying	74.6 (28.1)	70.0 (27.5)	74.5 (25.4)	73.9 (27.0)	73.9 (24.4)	78.1 (27.5)	1.02	0.407
Intimacy	78.7 (16.8)	66.0 (22.9)	71.0 (23.9)	76.1 (17.3)	69.8 (18.5)	68.3 (23.7)	8.44	0.001

* QoL = Quality of Life;

† ANOVA-F;

‡ p-value

Table 3 shows the quality of life scores according to the living arrangement, after adjusting for the potential confounding variables: age, gender, number of morbidities, and social support. In the group of older adults who lived alone, a lower quality of life score was identified in the intimacy facet compared to those who lived only with their spouse (β=-0.087; *p*=0.013), regardless of age, gender, number of morbidities, and social support. The older adults who lived only with their children, on the other hand, had lower scores on functioning of the senses (β=-0.117; *p*=0.006), autonomy (β=-0.082; *p*=0.043), and intimacy (β=-0.079; *p*=0.022) in the comparison with the group that lived only with the spouse, even after adjusting for the other confounding variables.

**Table 3:** Quality of life scores according to older adults' living arrangements, after adjusting for the potential confounding variables: age, gender, number of morbidities, and social support. Uberaba, MG, Brazil, 2017-2018

Domains/Facets QoL^†^	Adjusted multiple linear regression model*				
	Alone	Children only	Spouse and others	Children and others	Other types
	β^‡^(*p*)^§^	β^‡^(*p*)^§^	β^‡^(*p*)^§^	β^‡^(*p*)^§^	β^‡^(*p*)^§^
*WHOQoL-Bref*					
Physical	0.038 (0.291)	0.001 (0.980)	-0.032 (0.362)	-0.046 (0.188)	0.013 (0.710)
Psychological	-0.009 (0.808)	-0.015 (0.678)	-0.024 (0.502)	-0.068 (0.055)	0.007 (0.850)
Social relationships	-0.038 (0.303)	-0.066 (0.076)	0.018 (0.613)	-0.032 (0.377)	-0.062 (0.072)
Environment	-0.010 (0.787)	0.019 (0.611)	-0.012 (0.746)	-0.052 (0.161)	-0.011 (0.764)
*WHOQoL-Old*					
Functioning of the senses	-0.005 (0.906)	-0.117 (0.006)	-0.005 (0.906)	-0.100 (0.015)	0.017 (0.676)
Autonomy	0.025 (0.545)	-0.082 (0.043)	-0.094 (0.019)	-0.056 (0.154)	-0.039 (0.309)
Past/Present/Future activities	-0.037 (0.328)	-0.014 (0.714)	-0.045 (0.220)	-0.085 (0.021)	-0.032 (0.365)
Social participation	-0.026 (0.511)	-0.031 (0.423)	-0.083 (0.032)	-0.112 (0.003)	-0.024 (0.507)
Death and dying	-0.023 (0.601)	0.028 (0.527)	-0.002 (0.961)	0.015 (0.718)	0.065 (0.120)
Intimacy	-0.087 (0.013)	-0.079 (0.022)	-0.019 (0.566)	-0.113 (0.001)	-0.114 (< 0.001)

* Reference category: spouse only;

† QoL = Quality of Life;

‡ Regression coefficient;

§ p-value

Also in relation to the reference group (only with the spouse), the older adults who lived with the spouse and others obtained lower scores in autonomy (β=-0.094; *p*=0.019) and social participation (β=-0.083; *p*=0.032); in the type of arrangement children and others, lower scores were identified in functioning of the senses (β=-0.100; *p*=0.015), past, present and future activities (β=-0.085; *p*=0.021), social participation (β=-0.112; *p*=0.003), and intimacy (β=-0.113; *p*=0.001); and, finally, in the group of other types, the lowest scores were observed in the intimacy facet (β=-0.114; *p*<0.001). It is noteworthy that these associations remained significant even after adjusting for the potential confounding variables (Table 3).

## Discussion

In this study, the highest percentage of older adults lived only with their spouse (22.2%), followed by those who lived with their spouse and others (20.0%) and alone (18.5%). Partly corroborating these findings, an investigation with data from the National Household Sample Survey (*Pesquisa Nacional por Amostra de Domicílios*, PNAD) identified that the most representative older adult living arrangements were the following: couple who lived with children and other relatives (24.8%); single parent (24.1%), in which the reference person was the child and/or other relatives; and couple with children (18.9%)^(^ 15 ^)^.

Regarding gender, other studies have also verified a predominance of older adult women living alone^(^ 3 ^,^ 6 ^,^ 15 ^)^, which can be explained by the higher life expectancy of women in Brazil^(^ 22 ^)^. In addition, men in a situation of widowhood or separation tend to remarry^(^ 15 ^)^; converging with the result of this study, where the highest percentage of older adults living with their spouse and others pertained to the male gender.

Regarding the age group, there was a higher percentage of older adults who lived alone aged between 70 and 80 years old. National data corroborate the findings of the present study by showing a prevalence of older individuals (75 years old or more) living in single-person homes^(^ 6 ^)^. A research study carried out in Canada also identified a higher proportion of older adult people living alone compared to the others, in both genders^(^ 4 ^)^. Over time, older adults become more susceptible to living alone due to widowhood. In Brazil, the estimated life expectancy of the population in 2017 was 76 years old^(^ 23 ^)^.

Although the number of surviving generations has increased due to the increase in life expectancy, there has been a weakening of the ability of families to provide care for the older adults due to several factors: reduction in the fertility rates, greater insertion of women in the labor market, and presence of ageism in modern society^(^ 1 ^)^.

It should be mentioned that the increase in the older adults’ single-person homes represents an indicator of a successful aging process, as they can experience this moment independently and autonomously^(^ 3 ^)^. However, over the years, the risk of functional impairment increases^(^ 14 ^,^ 24 ^)^, which can hinder access to the health services and the performance of daily tasks^(^ 3 ^)^.

With regard to schooling, a similar result was evidenced in a study carried out using data from the National Health Survey, in which the majority of the Brazilians older adults reported having elementary education or lower (76.7%)^(^ 25 ^)^. The schooling level eventually interferes in the health-disease process, in the search for health services, and in adherence to treatments^(^ 26 ^)^. Consequently, older adults who live alone and have a low level of schooling can be more vulnerable to worsening health conditions compared to those who live with a partner or who have more years of study^(^ 6 ^)^.

In this study, there was predominance of older adults who received a minimum wage in all types of living arrangements. As age advances, health expenditures can increase, especially for the purchase of medications^(^ 27 ^)^, with the risk of compromising the basic needs of the older adults who receive this monthly amount. This result is of particular concern for the older adults who live alone, since a nationwide study with data from the Family Budget Survey identified that older adults in single-person homes commit 63.38% of their income to meet basic needs such as housing, health care and food at home^(^ 28 ^)^. According to the authors, the commitment of income affects the availability of resources for leisure activities and other essential expenses^(^ 28 ^)^, which can compromise quality of life for the older adults in this housing condition.

With regard to the comparison of the QoL scores with living arrangements in the bivariate analysis, previous findings corroborate the results obtained in the present study, as they indicate that cohabitation with the spouse was positively related to the QoL of older adults^(^ 5 ^,^ 7 ^)^ or because they show that living alone or with their children/others resulted in worse assessments^(^ 9 ^,^ 12 ^-^ 13 ^,^ 29 ^)^. However, these surveys did not adjust for social support, which was analyzed in the current study.

It is known that the type of living arrangement can interfere in the social interactions of the older adults and in the provision of available resources in their daily lives^(^ 7 ^)^. A study carried out with older adults who lived alone in South Korea showed that social support was a determining variable for the QoL domains^(^ 30 ^)^. According to the authors, the older adults in this housing context are minimally dependent on their families, as they have learned to manage everyday problems independently.

However, the national and international literature has reported that the older adults who live with their partner have better levels of physical and mental health^(^ 2 ^,^ 7 ^)^. It is possible that the spouse, for also experiencing the aging process, has more empathy with their partner and, together, create bonds of mutual support in daily life, overcoming the limitations imposed by old age. Social interaction in the home environment between the older adults and their partner can also avoid social isolation, as well as providing affective and material support. Thus, the social support network of older adults who live with their spouse can favor better QoL scores in this group, due to the mediating role between these variables.

Concerning the adjusted analysis, the living arrangement groups without cohabitation with the partner had lower scores in the intimacy facet compared to those who lived only with the spouse, regardless of age, gender, number of morbidities, and social support. A study carried out in the macro-region of the Southern Triangle, in Minas Gerais, obtained similar results, as older adults who lived without a partner also achieved lower scores in this facet^(^ 31 ^)^. Another study developed with urban and rural older adults in this study’s municipality verified that the absence of a partner was an independent predictor of the lowest QoL scores in the intimacy facet^(^ 32 ^)^.

In contemporary society, although families encourage the older adults to have moments of leisure and fun, there is no family support for them to experience new romantic relationships and exercise their sexual freedom after widowhood or marital separation^(^ 33 ^)^. A survey carried out in France with individuals aged 80 and over identified worse QoL scores in the dimension related to the sexual function. According to the researchers, being single and not living with a partner can negatively interfere in the sexuality of the older adults, especially women^(^ 34 ^)^. In addition, co-residence with the partner has contributed to the feeling of belonging and security, reducing the feeling of loneliness among the older adults^(^ 2 ^)^. It is therefore inferred that the absence of a partner can compromise the ability of the older adults to develop personal and intimate relationships, aspects assessed in this QoL dimension.

In general, there was a tendency for living arrangements with the presence of children and/or other family members to be negatively associated with the domains of autonomy and/or social participation in relation to the reference group (only with the spouse). Partially ratifying these findings, in a study developed in Turkey, the older adults who lived with other family members and without a spouse also had lower QoL scores in the domains of autonomy and social participation^(^ 13 ^)^. The social representations of the family about the older adult contribute to the stereotyped images of old age, relating them to inactivity, discouragement, and physical and cognitive decline. Consequently, there is an excess of paternity of the family towards the older adults, placing them in the role of passive beings and exempting their ability to make their own decisions and expand social life^(^ 35 ^)^.

Additionally, impairment, especially of the functioning of the senses, can contribute to the older adult choosing to live with other family members, especially children, since more weakened individuals are more prone to this type of arrangement^(^ 7 ^)^. A research study conducted with Chinese older adults in urban and rural areas revealed that single older adults living with adult children had worse physical health^(^ 5 ^)^. Equivalent data were found in this study, in which living only with children and others was associated with lower scores on functioning of the senses. Based on the assumption that this facet assesses the impact of the loss of sensory abilities on quality of life, it is believed that cohabitation with children and others is an alternative for older adults with some decline in the functioning of the senses during the aging process.

Also referring to the living arrangement made up of children and others, it was identified that the older adults in this group had a lower QoL score in the past, present and future activities facet in relation to those who lived with their spouse. In the older adults’ perception, old age is eventually linked to the end of life and to the lack of capacity to perform the same activities they performed when they were young^(^ 36 ^)^. In this regard, it is up to the children, formerly encouraged by their parents to build and implement projects throughout their lives, to encourage their parents, who now experience aging, to think about the future and plan it, taking into account what they yearned to live in the past, still yearn and have not experienced.

The main limitations of this research study are related to its cross-sectional design, which does not allow inferring a causal relationship between the predictor variables and the outcome. In addition, the study was carried out with a sample of older adults from a municipality in Minas Gerais, which makes it impossible to generalize the results to other regions of the country, mainly due to the possibility of cultural characteristics interfering in the configuration of living arrangements. Therefore, multicenter research studies, in particular with a longitudinal cut, are necessary to understand the impact of living arrangements on the quality of life of the older adults in different cultural contexts.

Despite this, the results evidenced in this research allow understanding the association between the living arrangement and the QoL domains and facets of a representative sample of urban older adults in the community, using a specific instrument for this age group and adjusting for potential confounding variables, such as social support.

In order to understand the specificities of each family context during the planning of care for the older adults, nurses from the Family Health Strategy can use different instruments, for example, the genogram and the ecomap, to identify the formal and informal resources available to the older adults and their families^(^ 3 ^)^. This understanding allows assessing the impact of the social and environmental conditions on the quality of life of the older adults, identifying possible protective and/or risk factors for their physical, psychological, mental, spiritual, and social health.

## Conclusion

This study revealed that the type of living arrangement was associated with QoL scores for older adults in the community, even after adjusting for the gender, age, number of morbidities, and social support variables. In relation to the group that lived only with the spouse, older adults who lived alone had a lower QoL score in the intimacy facet, and those who lived only with their children, lower QoL scores in functioning of the senses, autonomy, and intimacy.

Also in the comparison with the group that lived only with the spouse, older adults who lived with the spouse and others had a worse assessment of QoL in autonomy and social participation; those who lived with children and others, a worse assessment of functioning of the senses, past, present and future activities, social participation, and intimacy; and, finally, in the group of other types of living arrangement, a worse assessment in the intimacy facet.

Therefore, the findings of this research converge to the understanding that each modality of family composition has its strengths and weaknesses that need to be considered by researchers, educators, health and social care professionals and, above all, by policy-makers.
